# Prioritizing investments in new vaccines against epidemic infectious diseases: A multi‐criteria decision analysis

**DOI:** 10.1002/mcda.1683

**Published:** 2019-07-01

**Authors:** Dimitrios Gouglas, Kevin Marsh

**Affiliations:** ^1^ Norwegian Institute of Public Health Oslo Norway; ^2^ Epidemic Preparedness Innovations Oslo Norway; ^3^ Evidera‐Patient‐Centered Research London UK

**Keywords:** CEPI, epidemic infectious diseases, health research priority setting, multi‐criteria decision analysis, research and development, vaccines

## Abstract

**Background:**

In 2016, the Coalition for Epidemic Preparedness Innovations (CEPI) launched a call for proposals (CfP) for vaccine development against Lassa, MERS, and Nipah. CEPI is faced with complex decisions that involve confronting trade‐offs between multiple objectives, diverse stakeholder perspectives, and uncertainty in vaccine performance.

**Objective:**

This study reports on a multi‐criteria decision analysis (MCDA) and its testing on CEPI decisions.

**Methods:**

Consultations with CEPI's Scientific Advisory Committee (SAC) and document reviews helped identify and structure the criteria against which to evaluate proposals. Forty four subject‐matter experts assessed performance of 18 proposals on multiple criteria. SAC preferences were elicited via a survey employing an adapted swing‐weighting technique and were incorporated into measures of value and cost‐to‐value. A Monte Carlo simulation estimated overall value and ranking probabilities by value and by cost‐to‐value for each proposal.

**Results:**

Reviewer assessments and SAC preferences varied significantly. Despite this uncertainty, 14 preferred proposals emerged from the analysis and SAC recommendations on the basis of value and cost‐to‐value. In some cases, SAC recommendations deviated from the analysis because of: less emphasis on cost‐to‐value if budgets seemed underestimated by applicants, more emphasis on the likelihood of generating vaccines for target pathogens versus platform potential against unknown pathogens, and emphasis on funding a diversity of platforms per pathogen.

**Conclusions:**

Despite vaccine performance uncertainty and stakeholder preference heterogeneity, MCDA distinguished between options in a way that broadly corresponded to decisions. Divergence between the MCDA and the SAC point to potential updates needed to the model such as platform diversity trade‐offs.

## 1 INTRODUCTION

Following the successful vaccine research and development (R&D) response to the 2014 West‐African Ebola epidemic (Grobusch & Goorhuis, 2017), the World Health Organization (WHO) prioritized 11 epidemic infectious diseases (EIDs) most likely to cause severe outbreaks in the near future (WHO, 2016). Vaccines can prevent EID outbreaks from becoming humanitarian crises (CEPI, 2016a; Kieny et al., 2016). However, market incentives have failed to sustain R&D efforts in this area (Plotkin, 2016). A new entity, the Coalition of Epidemic Preparedness Innovations (CEPI), was set up in 2016 with a US$1 billion investment target to support the development of vaccines, contributing to the world's preparedness for EID outbreaks (CEPI, 2016a).

One of CEPI's first business plan targets was to advance the development of two to three vaccine candidates against priority EIDs from preclinical through to end of early clinical safety and immunogenicity testing (Phase IIa) by 2022 (CEPI, 2016a). By doing so, CEPI aimed to address the “just‐in‐case” R&D preparedness gap associated with lack of Phase IIb/III‐ready EID vaccines in advance of epidemic outbreaks.

Just like any funder of pharmaceutical R&D, CEPI is faced with the challenge of prioritizing limited resources in order to meet inherently risky R&D targets (Aurentz, Kirschbaum, & Thunecke, 2011). Evidence suggests that the average probability of successfully advancing a vaccine candidate from preclinical through to end of Phase II is less than 10% (Pronker, Weenen, Commandeur, Claassen, & Osterhaus, 2013). In addition, the large costs (DiMasi, Grabowski, & Hansen, 2016; Gouglas et al., 2018) and long timelines (Russell & Gronwall, 2012) involved in developing vaccines make investment decisions in this space tremendously complex. This complexity is compounded by the fact that where commercial objectives are lacking, such as in the field of EID vaccines, commercial value alone is an insufficient criterion to making pharmaceutical R&D investment decisions (Antonijevic, 2015; Cioffe, 2011; Perez‐Escobedo, Azzaro‐Pantel, & Pibouleau, 2012; Phillips & Bana e Costa, 2007).

To address its business plan targets in line with mission and scope, CEPI launched a competitive call for proposals (CfP) in late January 2017 to support the development of vaccine R&D in three priority EIDs: Lassa Virus, Middle East respiratory syndrome coronavirus (MERS‐CoV), and Nipah virus. The rationale was to invest in vaccine R&D projects that would improve the likelihood of generating vaccines relevant for use in response to these EIDs; as well as improve the likelihood that the platform technologies supporting these vaccines would be suitable for use in vaccine development against newly or unexpectedly emerging EIDs.

Evaluating proposals received in response to the call faces several challenges. First, pharmaceutical R&D portfolio management involves considering multiple criteria, including organizational capabilities, technical and manufacturing feasibility, development timelines and costs, and alignment with target product profiles (TPPs; Aurentz et al., 2011; Bode‐Greuel & Nickisch, 2008; Seget, 2005). For instance, the WHO has been advocating for use of ideal TPPs, or preferred product characteristics (PPCs) tailored to EID outbreak preparedness needs, to determine use potential of vaccines and to guide R&D priorities in the field (WHO, 2018).

Second, stakeholder opinions varied on the relative importance of different objectives. CEPI's Board has the ultimate decision‐making authority on all CEPI R&D investments. An independent, multi‐member Scientific Advisory Committee (SAC) advises the CEPI Board and Secretariat on R&D investments and makes technical recommendations for project funding. The composition of the SAC is diverse, and at the time of the deliberations described here included nine representatives of governments and regulators, seven industry members, eight academics, and four representatives of non‐profit R&D organizations (CEPI, 2016b).

In this context of multiple trade‐offs and heterogeneous stakeholder perspectives, multi‐criteria decision analysis (MCDA) has the potential to improve the quality of decision making (Marsh et al., 2016; Viergever, Gouglas, & Tromp, 2017). MCDA has become increasingly popular in health valuation (Marsh et al., 2017; Thokala et al., 2016) and its applications are numerous across a variety of areas in health (Adunlin, Diaby, & Xiao, 2015) and associated decision problems (Drake, de Hart, Monleon, Toro, & Valentim, 2017; Marsh et al., 2017; Thokala et al., 2016). MCDA can offer a rational and transparent approach to priority setting, simultaneously considering all relevant criteria to avoid ad hoc decisions (Baltussen & Niessen, 2006). Where there are multiple stakeholders with diverse perspectives, MCDA can make relevant conflicts explicit, helping decision makers understand them and consider their impact on decisions (Phillips & Bana e Costa, 2007; Timmis, Black, & Rappuoli, 2017).

The use of MCDA has been increasingly advocated in vaccine R&D (Barrochi, Black, & Rappuoli, 2016; Timmis et al., 2017). However, to our knowledge, only one other MCDA framework has been applied to support the prioritization of vaccine R&D (see for instance Phelps et al., 2014; Kloeber, 2011; Madhavan et al., 2012; Madhavan et al., 2013; Madhavan et al., 2015). This framework places emphasis on different attributes of burden of disease, which are difficult to apply in the CEPI context, given the sporadic and unpredictable nature of EIDs. Moreover, it does not lend itself easily to the estimation of value of vaccine R&D, which is adjusted for the probability of success (PoS) of early stage, risky vaccine candidates; nor does it assume sources of preference and constraints that are relevant to the CEPI decision context.

An MCDA framework was developed in accordance with ISPOR Good Practice guidelines (Marsh et al., 2016) to inform the prioritization of EID vaccine R&D proposals and support CEPI CfP decisions and was tested against the SAC recommendations. This study reports on the application of the MCDA framework. Results are anonymized because of confidentiality restrictions associated with ongoing contract negotiations between CEPI and developers of selected proposals for funding.

## 2 METHODS

The analysis focused on 18 full proposals that were selected by the CEPI SAC for an extended review following on an initial review of 33 preliminary proposals (CEPI, 2017a). The 18 proposals had a combined budget of over US$700 million and were reviewed by CEPI between March and May 2018. Seventeen proposals were at the preclinical development phase and one proposal was at clinical phase 1, with the aim that CEPI funding would advance them to the end of clinical phase 2. Proposals covered three different types of diseases: Lassa, MERS, and Nipah. Proposal budgets ranged from US$22 million to US$68 million, with a median cost of US$35 million. Proposal timeframes through to end of clinical phase 2 ranged from 4 to 6 years, with a median timeframe of 5 years. Due to confidentiality restrictions, individual proposal budgets and timeframes are not reported here; however, it is these budgets and timeframes that have been used to generate values in the framework presented below. Proposal names and disease classifications have been anonymized throughout the remainder of this manuscript. Proposals have been labelled as P1 to P18 and platform types are labelled 1–3. Seven proposals covered disease 1; seven proposals covered disease 2; and four proposals covered disease 3.

The goal was to undertake a quantitative valuation and ranking of the 18 proposals against criteria that were of interest to the SAC. It was assumed that not more than 14 proposals could be funded, given the resources available. The remainder of this section provides a step‐by‐step overview of the modelling approach adopted (more details provided in Data S1).

Step 1. Value framework

Between October 2016 and December 2016, a long list of potential value criteria was initially generated via document reviews, including: the CEPI Business Plan (CEPI, 2016a); documents from CEPI consultations informing the business plan (Røttingen et al., 2017); CEPI policy documents on principles of equitable access, cost coverage, risk sharing, and management of intellectual property (CEPI, 2017b); the WHO Blueprint process (WHO, 2016); evaluation criteria used by other agencies of health R&D funding in Europe and the United States—such as Biomedical Advanced Research and Development Authority (BARDA, 2018), Innovative Medicines Initiative (IMI, 2018), Horizon 2020 (EC, 2018), and national aid agencies supporting Product Development Partnerships active in global health R&D (DFID, 2015; Gouglas & Plahte, 2015; NEA, 2018). Additional contributions to this list came from semi‐structured interviews with 19 members of the SAC, which were conducted in parallel with the document review process.

**Table 1:** Criteria CfP vaccine development Lassa‐MERS‐Nipah

Criterion	Metric	Assessment informed by:
C1. Applicant competencies, experience & track‐record	Overall likelihood that the applicant is sufficiently competent to deliver on the proposed activities of the project (0–100%)	• Technical competency/expertise of project staff • Experience in preclinical testing of vaccines • Experience in conduct of Phase I/II clinical vaccine trials • Experience in regulatory interactions with competent authorities and licensing of vaccines • Manufacturing capabilities and skills
C2. Technical feasibility	Overall likelihood that the development of the candidate vaccine through phase II is technically feasible (0–100%)	• Soundness of the theoretical concept/scientific rationale • Quality of the integrated product development plan • Current development status/technical readiness • Soundness of the clinical development and regulatory approach
C3. Manufacturing scalability & speed	Overall likelihood that the vaccine candidate is manufacturable and scalable in timeframes and volumes to respond to outbreaks (0–100%)	• Soundness/scientific rationale of manufacturing processes/technologies supporting the candidate vaccine • Current status/availability of manufacturing • Manufacturing capacity and yield • Time to produce/release sufficient quantities of vaccine for emergency use in response to a disease outbreak • Suitability of manufacturing processes/technologies for large scale production and delivery in an emergency
C4. Use potential for target pathogens	Overall likelihood that the candidate vaccine will meet CEPI's ideal Target Product Profile and, if not, that any deviations from this will be still relevant for use of the vaccine in emergency (0–100%)	• Suitability of the candidate vaccine for outbreak control • Suitability of the candidate vaccine for routine use
C5. Use potential for new pathogens	Overall likelihood that the platform technology supporting the candidate vaccine(s) will be suitable for use in vaccine development against newly emerging/unexpected pathogens (0–100%)	• Suitability of the technology platform for other pathogens of the WHO priority list of emerging infectious diseases • Suitability of the technology platform for other pathogens beyond the WHO priority list of emerging infectious diseases
O1. Likelihood of generating a suitable vaccine for one of the CfP target pathogens	Overall likelihood that the project will generate a vaccine that is relevant for use in response to one of the CfP target pathogens (0–100%)	• Probability of successful vaccine development from preclinical through phase II (criteria C1 to C3) times the probability of use for CfP target pathogens (C4)
O2. Likelihood that the platform technology will be suitable for vaccine development against new pathogens	Overall likelihood that the platform technology supporting the vaccine will be suitable for use in response to newly emerging and/or unexpected pathogens (0–100%)	• Probability of successful vaccine development from preclinical through phase II (criteria C1 to C3) times the probability of use for new pathogens (C5)

To narrow down the list of criteria, and combine the criteria into a value framework, members of the SAC and CEPI secretariat staff were asked first in an email survey and then in a group discussion in January 2017 to determine: whether all factors relevant to CfP decisions had been captured by the criteria; the relationship between the criteria, and whether any of the criteria should be removed or re‐grouped if overlapping, or irrelevant. Following this engagement, overall value ( *Vi* ) was estimated as described in Equation (1).

(1) $$V_{i}=\frac{1}{{\left(1+r\right)}^{t_{i}}}.\left(W_{O1}.{\mathrm{PV}}_{O1}.O1_{i}+W_{O2}.{\mathrm{PV}}_{O2}.O2_{i}\right)$$

Where:

V_i_ = overall value of proposal i.

O1_i_ = likelihood of generating a suitable vaccine for one of the CfP target pathogens.

O2_i_ = likelihood that the platform technology will be suitable for vaccine development against new pathogens.

W_O1_ = weight given to likelihood of generating a suitable vaccine for one of the CfP1 target pathogens.

W_O2_ = weight given to likelihood that the platform technology will be suitable for vaccine development against new pathogens.

PV_O1_ = partial value function for likelihood of generating a suitable vaccine for one of the CfP1 target pathogens.

PV_O2_ = partial value function for likelihood that the platform technology will be suitable for vaccine development against new pathogens.

t_i_ = timeframe over which the proposal i will deliver.

r = discount rate.

A number of other criteria were identified as defining a proposal's performance against *O*1 and *O*2. Equations 2 and 3 describe how performance against these criteria were combined multiplicatively to estimate *O*1 and *O*2 for each proposal (*i*). Criteria C1 to C5 are defined in Table 1. Each of these criteria is defined as a probability on a measurement scale 0–100%.

(2) $$O1_{i}=C1_{i}.C2_{i}.C3_{i}.C4_{i}$$

(3) $$O2_{i}=C1_{i}.C2_{i}.C3_{i}.C5_{i}$$

where:

C1_i_ = experience and track‐record: Likelihood that the applicant is sufficiently competent to deliver on the proposed activities of the project, for a given proposal *i*.

C2_i_ = feasibility: Likelihood that the development of the candidate vaccine through phase II is technically feasible, for a given proposal *i*.

C3_i_ = manufacturing scalability and speed: Likelihood that the vaccine candidate is manufacturable and scalable in timeframes and volumes to respond to outbreaks, for a given proposal *i*.

C4_i_ = use potential for CfP target pathogens: Should a vaccine candidate be successfully developed and manufactured, the likelihood that it will meet CEPI's Target Product Profile and will be relevant for use in an emergency, for a given proposal *i*.

C5_i_ = use potential for new pathogens: Should a vaccine candidate be successfully developed and manufactured, the likelihood that the platform technology supporting the candidate vaccine will be suitable for use in vaccine development against newly emerging pathogens for a given proposal *i*.

The value framework presented in Equations 1–3 was presented to and approved by the SAC in February 2017, together with proposed criteria descriptions, measurement scales, and appraisal questions for reviewers (see Table 1).

Criteria C1, C2, and C3 presented above relate to the probability that the vaccine candidate and the technology platform supporting its development can be successfully advanced through to end of clinical phase 2. Criteria C4 and C5 relate to the anticipated benefits from these proposals, if successfully developed through to end of clinical phase 2. Specifically, criterion C4 relates to the anticipated clinical and operational benefits of the vaccine candidate in response to an outbreak of the targeted disease, if the candidate vaccine was to be successfully developed through to end of clinical phase 2. Criterion C5 relates to the anticipated potential of the technology platform used to develop the candidate vaccine to support the development of other candidate vaccines against newly or unexpectedly emerging pathogen outbreaks, regardless of whether the development of the vaccine candidate against the currently targeted pathogen was successful or not.

Assuming a technology platform is successfully developed, the value of its potential to be used to develop a vaccine against a targeted pathogen (C4) is not dependent on its potential to be used to develop a vaccine against an unknown pathogen (C5) and vice versa—they are additively valuable. However, for either of these potentials to be realized, the platform needs to be successfully developed, which is reflected in criteria C1–C3. Moreover, there is no value in a platform being technically feasible (C2), if the vaccine developer does not have the competency to develop it (C1), vaccines cannot be manufactured to scale on this platform (C3) or the platform does not have the potential to support vaccine development against a pathogen (C5)—so these criteria were combined multiplicatively.

Step 2. Measuring performance (C1_i_,C2_i_,C3_i_,C4_i_,C5_i_ )

Proposals were assessed against criteria C1 to C5 by 44 external reviewers with subject matter expertise on EID vaccine development and no conflicts of interest. Reviewers were selected through an open competitive process on the basis of demonstrable experience—including years of work experience—in non‐clinical, clinical, chemistry, manufacturing, and control aspect of vaccine development. Each proposal was assessed by three to five reviewers. Reviewers received a manual and presentation providing detailed descriptions of criteria, scorecard templates, instructions, and examples for filling in these templates. Further assistance and clarifications were provided in response to specific questions over email and phone throughout the review process.

For each criterion C1–C5, reviewers were asked to define the most likely worst‐case and best‐case performance of proposals on a scale of 0–100% (see Data S1 for details). In order to determine the degree of homogeneity in the assessments provided by the different reviewers, an inter‐reviewer assessment variability test was conducted. Specifically, for each criterion C1–C5, and for each performance estimate (worst‐case, most likely, best‐case), the following steps were undertaken. First, the performance mean across all reviewers assessing a given proposal was calculated. Second, the difference between this mean and each reviewer's performance estimate on the given proposal was calculated. Third, steps 1 and 2 were repeated for all proposals. Fourth, for each reviewer, the average deviation of his or her performance estimate from the performance mean across all of his or her assessed proposals was estimated. Fifth, on the basis of Cicchetti's (1994) classification, reviewer variability was determined as good if this average deviated less than 20% from the performance mean, and excellent if it deviated less than 10% from the performance mean across all of his or her assessed proposals. Seven of the 44 reviewers were found to have at least one average worst‐case, most likely, or best‐case performance estimate against C1–C5 that deviated more than 20% from the equivalent performance mean across all their assessed proposals. In total, the estimates for which such deviations were observed accounted for only 3% of the total number of worst‐case, most likely, and best‐case performance estimates collected. The impact of removing these results from the analysis was tested and found to not substantially change the performance of proposals.

Step 3. Estimating partial values (PV_O1_ , PV_O2_ )

**Figure 1:**
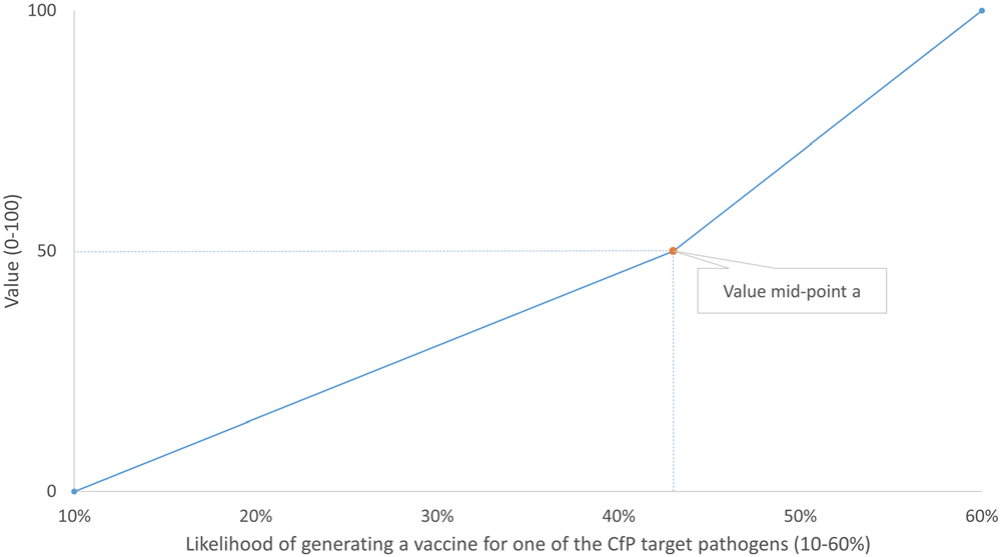
Partial value function of likelihood of generating a vaccine for one of the CfP target pathogens: an illustration

Partial value functions were elicited for *O*1 and *O*2 from each SAC member using an online survey (24 respondents out of 29 survey recipients). The functions were defined using a mid‐value splitting method—a widely‐used decomposed scaling technique also known as the bi‐section method (Von Winterfeldt & Edwards, 1986)—by eliciting the value mid‐point on a 10%–60% performance range (point a in Figure 1).

In the questioning procedure applied for the elicitation of partial values on each of *O*1 and *O*2, SAC members answered up to six pairwise choice questions that iteratively approached this value mid‐point. For instance, for *O*1 the first question was:

“Consider the following two proposals, each with different starting likelihoods of generating a vaccine that will be relevant for use in response to one of the CfP target pathogens. Imagine you are given the opportunity to improve the performance of one of these proposals. Which of the following options would you prefer?

- Option A: Improve Proposal A so that the likelihood that it generates a vaccine that will be relevant for use in response to one of the CfP target pathogens increases from 10% to x%
- Option B: Improve Proposal B so that the likelihood that it generates a vaccine that will be relevant for use in response to one of the CfP target pathogens increases from x% to 60%
- Indifferent between options A and B″

In the first question, *x* was set as the mid‐point in the performance range (35%). If a respondent was indifferent, the partial value function was considered linear, and no further questions were asked. If a respondent chose option A or option B, the value of *x* was updated according the logic defined in Section 2 of the Data S1.

The pairwise choice questions identified *a* to be within a range. It was assumed that *a* was the mid‐point in this range, on the basis of which partial value functions could then be defined (see Section 2 of Data S1).

Step 4. Estimating weights (W_O1_ , W_O2_ )

Weights were elicited for *O*1 and *O*2 using the trade‐off method (von Winterfeldt and Edwards, 1986), in each case for a range of performance of 10%–60%, from each SAC member using an online survey (24 respondents out of 29 survey recipients). An iterative pairwise comparison was used to identify the value of *b*, such that respondents would be indifferent between improving *O*1 from 10% to *y*% and improving *O*2 from 10% to 60%. Specifically, the following question was asked via an online survey:

“Considering the following two proposals, which of these would you prefer?

- Proposal A
  ○ Likelihood of generating a vaccine that will be relevant for use in response to one of the CfP1 target pathogens = y%
  ○ Likelihood that the technology will be suitable for use in vaccine development against newly emerging/unexpected pathogens = 10%
- Proposal B
  ○ Likelihood of generating a vaccine that will be relevant for use in response to one of the CfP1 target pathogens = 10%
  ○ Likelihood that the technology will be suitable for use in vaccine development against newly emerging/unexpected pathogens = 60%”

The initial value of *y* was set at 35%, and varied depending on responses as defined in Section 2 of the Data S1. After six questions, the value of *b* was defined within a range defined in Section 2 of Data S1. It was assumed that the value of *b* was the mid‐point in this range. Section 2 of the Data S1 provides more details on how b was used to estimate weights for *O*1 and *O*2.

Step 5. Eliciting time preference (r)

Time preference was estimated using a choice exercise designed to identify the value of *c*, such that SAC members were indifferent between a *z*% chance of successfully delivering a proposal within 5 years, and a 100% chance of doing so within 10 years. The following question format was implemented with SAC members in an online survey:

“Considering the following two proposals, which of these would you prefer?

- Proposal A
  ○ Time‐to‐completion = 5 years
  ○ Likelihood of successful completion = z%
- Proposal B
  ○ Time‐to‐completion = 10 years
  ○ Likelihood of successful completion = 100%”

The value of *z* in the first question was set at 55%, and then varied depending on responses in a manner described in Section 2 of Data S1. After up to six questions, the value of *c* was identified within a range described in Section 2 of Data S1. It was assumed that *c* took the value of the mid‐point in this range. Section 2 of the Data S1 provides more details on how c was used to estimated to estimate the discount rate.

Step 6 Dealing with uncertainty

Both reviewer performance inputs and SAC preferences were subject to significant variations. This uncertainty was incorporated into the MCDA via Monte Carlo simulation. The model was run 10,000 times, each time drawing from the different inputs, as follows:

- Performance inputs: For criteria C1 to C5, each iteration randomly selected one reviewer and randomly selected a performance estimate from their performance distribution.
- SAC preferences: Each iteration randomly drew the partial value, weights, and time preference of a single SAC member's distributions.

The mean and 95% confidence intervals of performance on *C1–C5*, *O1*, *O2*, and *V* were estimated for each iteration of the simulation. Comparison of proposals within each iteration allowed a ranking of proposals, which, when analysed across all iterations, allowed the estimation of the rank probability of a proposal.

Step 7: Reporting the MCDA

Various iterations of the model were presented to the SAC over email and teleconferences for validation of its practical utility between December 2016 and July 2017. A detailed methodology document was shared in July 2017 and the model findings were presented during the SAC decision meeting in August 2017.

## 3 RESULTS

Criteria performances of the 18 vaccine R&D proposals are presented in Table 2. The uncertainty in performance means that there is substantial overlap in the confidence intervals around most proposals' performance on: the likelihood of generating a suitable vaccine for one of the CfP target pathogens (*O1)* and on the likelihood that the platform technology will be suitable for vaccine development against new pathogens (*O2)*.

**Table 2:** Proposal performances on criteria C1 to C5 (Mean, 95% CI)*, ^

		C1	C2	C3	C4	C5	O1	O2
**Disease 1**	**Proposal 13**	94% (90–100%)	87% (75–100%)	93% (75–100%)	81% (60–100%)	89% (80–100%)	62% (40–86%)	68% (51–90%)
	**Proposal 1**	92% (80–100%)	68% (25–95%)	88% (65–100%)	82% (50–100%)	81% (30–100%)	45% (14–81%)	45% (11–81%)
	**Proposal 17**	91% (85–100%)	73% (60–85%)	81% (60–100%)	81% (65–90%)	69% q(35–100%)	44% (24–65%)	37% (15–72%)
	**Proposal 10**	91% (80–100%)	76% (55–100%)	78%(45–100%)	82% (45–100%)	81% (35–100%)	39% (14–77%)	44% (16–81%)
	**Proposal 16**	77% (40–100%)	68% (30–100%)	78% (40–100%)	60% (15–100%)	86% (60–100%)	25% (3–77%)	35% (8–81%)
	**Proposal 8**	83% (55–100%)	58% (40–85%)	71% (50–95%)	68% (40–90%)	77% (45–100%)	23% (8–48%)	26% (9–54%)
	**Proposal 5**	78% (50–95%)	38% (20–55%)	65% (20–95%)	58% (15–90%)	78% (60–95%)	11% (2–29%)	15% (4–32%)
**Disease 2**	**Proposal 14**	93% (85–100%)	87% (70–100%)	93% (75–100%)	83% (60–100%)	89% (80–100%)	62% (39–86%)	66% (48–86%)
	**Proposal 3**	86% (70–95%)	73% (65–90%)	84% (60–100%)	66% (50–95%)	78% (65–95%)	35% (20–58%)	41% (25–61%)
	**Proposal 6**	83% (55–100%)	79% (50–100%)	71% (50–95%)	75% (65–90%)	77% (45–100%)	35% (15–61%)	35% (14–69%)
	**Proposal 12**	89% (65–100%)	81% (65–100%)	60% (40–90%)	71% (60–80%)	76% (60–95%)	31% (15–55%)	33% (16–61%)
	**Proposal 2**	83% (75–90%)	70% (50–85%)	67% (30–95%)	63% (35–95%)	73% (45–90%)	25% (7–51%)	28% (9–51%)
	**Proposal 15**	82% (65–90%)	61% (30–80%)	70% (45–95%)	62% (35–75%)	70% (40–90%)	22% (7–40%)	24% (9–46%)
	**Proposal 11**	69% (35–95%)	55% (20–75%)	77% (45–100%)	62% (40–100%)	69% (40–100%)	18% (4–47%)	20% (5–50%)
**Disease 3 proposals**	**Proposal 9**	89% (70–100%)	81% (65–100%)	82% (55–100%)	83% (65–100%)	67% (50–90%)	49% (27–77%)	40% (21–68%)
	**Proposal 18**	91% (85–100%)	73% (40–100%)	76% (40–100%)	72% (40–95%)	62% (30–100%)	37% (9–81%)	32% (5–86%)
	**Proposal 7**	83% (55–100%)	72% (60–90%)	72% (55–95%)	76% (65–90%)	77% (45–100%)	32% (17–51%)	33% (15–58%)
	**Proposal 4**	78% (50–95%)	38% (20–55%)	66% (20–100%)	46% (25–65%)	78% (60–95%)	9% (2–22%)	15% (4–34%)

* Proposals listed in order by disease, by O1 mean performance.

^ C1: Experience & track‐record; C2: Feasibility; C3: Manufacturing scalability & speed; C4: Use potential for CfP target pathogens; C5: Use potential for new pathogens; O1: Likelihood of generating a suitable vaccine for one of the CfP target pathogens; O2: Likelihood that the platform technology will be suitable for vaccine development against new pathogens

Table 3 presents the results of the preference elicitation survey. Greater weight was attached to performance on *O1* than *O2* by 92% of participants. The remaining participants gave equal weight to performance on *O1* and *O2*. Participants' discount rate was high, with 63% having a rate above 20%. Most participants' responses to the preference survey implied that the partial value function of both *O1* and *O2* was non‐linear with increasing marginal returns (54% for *O1* and 78% for *O2*). Though a small proportion of participants' responses implied a linear function, 29% and 13% for *O1* and *O2*, respectively, or decreasing marginal returns, 17% and 8% for *O1* and *O2*, respectively.

**Table 3:** Preference elicitation findings*

	O1 weight	O2 weight	O1 value mid‐point	O2 value mid‐point	Time discount rate
Mean	0.72	0.28	0.40	0.43	0.22
Standard Deviation	0.13	0.13	0.12	0.09	0.11
Lowest estimate	0.50	0.10	0.17	0.22	0.04
Highest estimate	0.90	0.50	0.60	0.60	0.46

* O1: Likelihood of generating a suitable vaccine for one of the CfP target pathogens; O2: Likelihood that the platform technology will be suitable for vaccine development against new pathogens.

**Figure 2:**
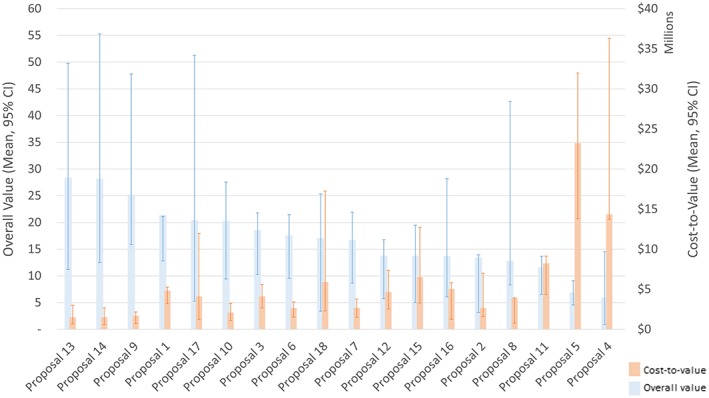
Proposal overall value and cost‐to‐value

Figure 2 presents the overall, discounted value and cost‐to‐value of the 18 proposals. Ranking of proposals was similar by overall value and cost‐to‐value, with the exception of a handful of proposals which had high budgets. Uncertainty in performance scores and preferences mean that there is substantial overlap in the confidence intervals around proposals' overall value. Over 90% of the variance observed in Figure 2 is explained by the variation in reviewer assessments of proposal performance.

**Figure 3:**
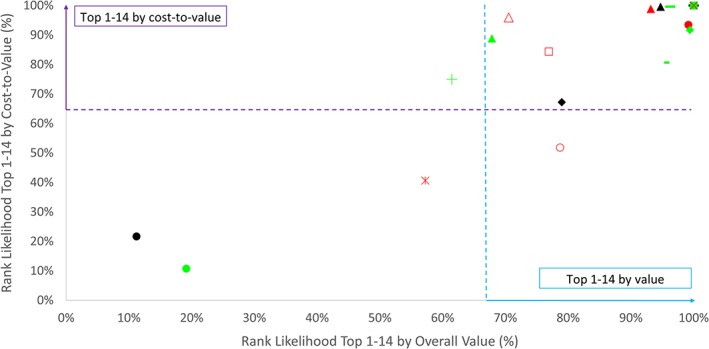
Top 1–14 ranking likelihood of proposals by overall value and by cost‐to‐value

Assuming that not more than 14 projects can be selected, Figure 3 plots the likelihoods of proposals ranking in the top 1–14, on the basis of discounted value versus cost‐to‐value. The consideration of budgets did not affect most of these ranking outputs in the analysis, with the exception of two proposals that substituted each other in the top 1–14 depending on whether they were ranked by value or by cost‐to‐value (top 1–14 by value to the right of the blue dotted line; top 1–14 by cost‐to‐value to the top of the purple dotted line, in Figure 3).

Figure 3 demonstrates that despite the large uncertainty in criteria performance and stakeholder preferences, clear proposal rankings emerged through the consideration of top 14 ranking likelihoods. SAC recommendations marginally deviated from these rankings. In a face‐to‐face meeting in August 2017, the SAC was presented with the reviewer assessments of each proposal and the results of the MCDA. Following a deliberation, they recommended 14 proposals for funding (proposals 1, 3, 4, 5, 6, 7, 8, 9, 10, 12, 13, 14, 17, and 18). Most of the recommended proposals had the highest probability of being ranked in the top 14 proposals by the MCDA on both value and cost‐to‐value. In some cases where SAC recommendations deviated from the analytical findings, the SAC's deliberation highlighted possible reasons for this divergence: A lower emphasis on cost‐to‐value as it was believed that some of the requested budgets were unrealistic and they could substantially increase during implementation; a lower emphasis placed on feasibility (*C2*) and manufacturing scalability and speed (*C3*) and a higher emphasis placed on use potential for new pathogens (*C5*). Additional considerations that contributed to the final selection recommendation included: a higher emphasis on target pathogens (*O1*) versus unknown pathogens (*O2*); and diversity consideration, in particular funding a diversity of platforms by CfP target pathogen.

**Figure 4:**
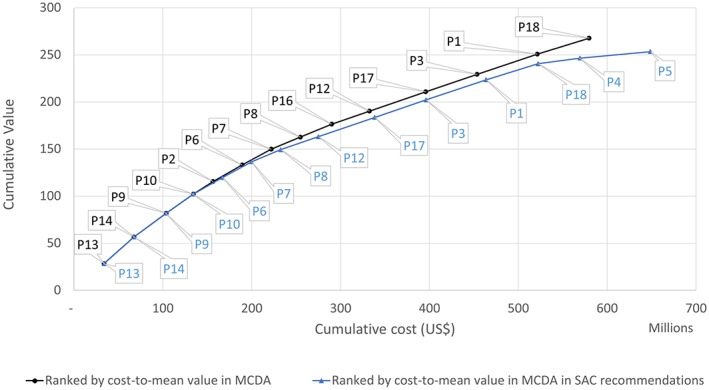
Efficiency frontier by different ranking methods

Figure 4 plots the top 14 ranked proposals by cumulative value and cumulative cost. These are ranked in two different ways, by: (a) cost‐to‐mean value and (b) proposals recommended for funding by the SAC by cost‐to‐mean value.

## 4 DISCUSSION AND CONCLUSIONS

This paper involved a number of innovations in the evaluation of early stage vaccine R&D candidates for EIDs, addressing gaps identified in previous literature. These included the explicit consideration of technical and operational feasibility of proposals using expert reviewer assessments of vaccine performance, in the absence of historical PoS data for these proposals; the multiplicative and additive combination of performance against a comprehensive list of criteria into an assessment of overall value of proposals, compliant with the theoretical properties required of a set of criteria in MCDA (Marsh et al., 2016) and applied as these properties have emerged in this particular decision context (Zeleny, 2011); the use of an adaptive swing weighting technique to elicit and incorporate stakeholder preferences into an assessment of overall value of proposals; and the use of Monte Carlo simulation to account for uncertainty in performance estimates and stakeholder preferences in proposal rankings.

CEPI's investment in EID vaccine R&D faced significant uncertainty in both the potential performance of proposed vaccine candidates—which were all in preclinical or early clinical phases of development—and stakeholder objectives. This is evident in both the MCDA model inputs and outputs, with substantial overlap in the confidence intervals on the overall value of proposals. Nevertheless, the use of a Monte Carlo Simulation reflected this uncertainty in rank probabilities that distinguished proposals, and that were broadly consistent with the SAC's recommendations.

During the SAC decision meeting in August 2017, MCDA findings informed deliberations on individual proposal performances and comparisons between proposals across diseases and platform technologies. The SAC's recommendations did not, however, correspond entirely with the MCDA. It was never the intention of the MCDA to remove the deliberative component of the decision‐making process. However, the divergence in SAC recommendations and model outputs point to some lessons from the research and was also a way to validate the practical usefulness of the model.

First, the SAC's deliberation pointed to criteria that could have been added to the MCDA, such as distributional considerations—spreading investment across proposals that employ different platforms. The implication of this was the selection of proposals that had modest value in terms of their combined performance across criteria *C1–C5* but which added desired platform diversity into CEPI's investment portfolio.

Second, the SAC placed less emphasis on cost‐to‐value, as in some cases proposed budgets were considered unrealistic. One implication of this was that some proposals that had small budgets but whose overall value was otherwise low were not prioritized.

Third, the SAC's deliberation pointed towards structural implications for the MCDA. A novel combined multiplicative‐additive model structure was adopted. Few MCDA applications in healthcare have multiplicative components (Marsh et al., 2016) despite concerns that health technology assessment does not meet the analytical requirements of additive models (Marsh, Sculpher, Caro, & Tervonen, 2018). The multiplicative component of the model implicitly gave equal weight to criteria *C1–C5*, whereas the SAC deliberation seemed to emphasize some of these criteria more than others (e.g., *C2* and *C4*).

Fourth, the SAC's recommendations could imply alternative weights to those used in the model; specifically that an even greater weight was given to *O1* than what was elicited through the survey. Weights in the model were elicited using an iterative comparison of improvements in pairs of criteria. This method was chosen due to the small sample size providing insufficient power for a discrete choice experiment; and a desire to elicit ordinal data in a survey format (Tervonen et al., 2017). The result was that, on average, more weight was given to *O1*. Though there was also significant variation in SAC member weights. Given this variation, one possibility that would reconcile the SAC recommendations with the result of the MCDA is that SAC members who gave a higher weight to *O1* were more influential in the deliberation.

In conclusion, the analysis reported in this study demonstrates that it is possible to use a MCDA to support the prioritization of vaccine R&D investments in a complex decision context characterized by outcomes uncertainty, variance in expected performance of vaccines, and heterogeneity of stakeholder preferences. With the intention to aid, rather than replace deliberative stakeholder processes or prescribe decisions, the findings illustrate how MCDA can help differentiate investments, and support decision making.

## CONFLICT OF INTEREST

Mr. Gouglas reports grants from Research Council of Norway, during the conduct of this study. Dr. Marsh reports grants and personal fees from Research Council of Norway and CEPI, during the conduct of the study.

## FUNDING

This work was partly supported by the Research Council of Norway through the Global Health and Vaccination Programme (GLOBVAC), project number 234608.

## ETHICAL APPROVAL

All procedures performed in studies involving human participants were in accordance with the ethical standards of the institutional and/or national research committee and with the 1964 Helsinki declaration and its later amendments or comparable ethical standards. This article does not contain any studies with animals performed by any of the authors.

## INFORMED CONSENT

Informed consent was obtained from all individual participants included in the study.

## Supporting information

Data S1:
Supplementary Material
