# Supplementary material for: Prioritizing investments in new vaccines against epidemic infectious diseases: A multi‐criteria decision analysis
Source: J Multi Criteria Decis Anal. 2019 Jul 1;26(3-4):153–63. doi: 10.1002/mcda.1683 (PMC7168397; doi:10.1002/mcda.1683)
Supplement: Supplementary file 1 — Data S1: Supplementary Material [file MCDA-26-153-s001.docx]

# **Appendix**

*Article title*: Prioritizing investments in new vaccines against epidemic infectious diseases: a multi-criteria decision analysis

*Journal name*: Journal of Multi-Criteria Decision Analysis

*Author names*: Dimitrios Gouglas (corresponding author), Kevin Marsh

*Affiliation and e-mail address of the corresponding author*: Norwegian Institute of Public Health/ Coalition for Epidemic Preparedness Innovations; Postal address: P.O. BOX 123, Torshov, 0412 Oslo; Visiting address: Marcus Thranes Gate 2, Oslo, Norway; Email: [dimitrios.gouglas@cepi.net](mailto:dimitrios.gouglas@cepi.net); Telephone: (+47) 98657142

This document provides supplementary detail on: (1) the methodology used to define performance distributions for assessing proposals (Section 1); and (2) the performance levels used in the elicitation questions (Section 2).

## Section 1. Defining performance distributions for assessing proposals

Reviewers’ performance estimates were collected online using the CfP review platform at the Research Council of Norway. This posed the practical limitation that reviewer online submissions were required to use a 1-7 points scale whereas reviewers were required to define the most likely, worst-case and best-case performance of proposals on a scale of 0-100%. The approach described below was therefore used to address the practical limitations of the Research Council of Norway’s submission system, in a way that would not impact the analytical objectives of this exercise.

Reviewers were asked to define the range of likely performance (%) of proposals on each criterion. Then assuming the worst-case was a score of 1 and the best-case was a score of 7, to give the most likely performance on the 1-7 scale.

A performance distribution was defined for each reviewer by following a number of steps:

- **Step 1.** Take the performance range and the most likely score (e.g. 60-100% with score 5)
- **Step 2.** Allocate this performance across a distribution of equal 5% blocks (see columns ‘Block distribution’ and ‘Performance Range’ in Table 1.1)
- **Step 3.** Assume a 100% probability that the performance falls within the performance range (see column ‘Total Probability’ in Table 1.1).

**Step 4.** Identify the ‘most likely’ performance block (the mode) as described in the following equation:

$B_{mode}= B_{L}+\frac{\left( B_{H}- B_{L} \right)}{S_{max}} * S_{mode}$

Where:

$B_{mode}$ = Most likely performance [rounded to the nearest 5% block]

$B_{L}$ = Lowest performance block

$B_{H}$ = Highest performance block

$S_{max}$ = Maximum score that can possibly be allocated, which is 7 according to criteria definitions

$S_{mode}$ = Most likely score

E.g. for a performance 60-100% (score 5):

$B_{mode}$ = 60% + $\frac{100\%-60\%}{7}*5$ = 88.6% [rounded up to 90%, the nearest 5% block]

**Step 5.** Allocate a probability of being within the mode block as described in the following equation:

$$p_{mode}=\left\{ \begin{aligned} 100\%, if N=1 \\ 50\%, if N>1 \end{aligned} \right.$$

Where:

$N= Total number of performance blocks=1+\frac{\left( B_{H}- B_{L} \right)}{5\%}$ $p_{mode}$ = probability of performance being within the mode block

E.g. for a performance of 60-100%:

$N= 1+\frac{\left( 100\% - 60\% \right)}{5\%}$ = 9

Therefore $p_{mode}$ = 50%, given that N = 9 is greater than 1

**Step 6.** Allocate the remaining probability to the remaining blocks as described in the following equation:

$${p(B)n}_{i}=\left[ \frac{100\%- \sum\left( X{p(B)n}_{i-1}+ {Xp(B)n}_{i-2}+\ldots+ {Xp\left( B \right)n}_{i-j}+ p_{mode} \right)}{N-({Xn}_{i}-Z)} \right]*2$$

Where:

*N* = Total number of performance blocks

$n_{i}$ = nth block deviation from $B_{mode}$

$$X=\left\{ \begin{aligned} 2, &if on the nth block deviation from B_{mode} there is a mirroring block on both sides of the distribution \\ 1, &if otherwise \end{aligned} \right.$$

$$Z=\left\{ \begin{aligned} 1, &if on the nth block deviation from B_{mode} there is a mirroring block on both sides of the distribution \\ 0, &if otherwise \end{aligned} \right.$$

$(B)n_{i}$ = Block in nth block deviation from $B_{mode}$

$B_{mode}$ = mode block

$p_{mode}$ = probability of performance being within the mode block

${p(B)n}_{i}$ = probability of performance being within block(s) of nth block deviation from $B_{mode}$

E.g. for a performance of 60-100%, with $B_{mode}$ = 90%, $p_{mode}$ = 50%, N = 9 the remaining 50% probability is allocated as shown in table 1.1:

**Table 1.1:** Example performance distribution (performance range 60-100%, most likely performance score 5)

| **Block distribution** | | **Performance Range** | **Total Probability** | | **Assigned Probability by performance block** | |
| --- | --- | --- | --- | --- | --- | --- |
| *0%* |  | | | *0% (for performance range 0-<60%)* | | 0% |
| *5%* |  | | |  |  | 0% |
| *10%* |  | | |  |  | 0% |
| *15%* |  | | |  |  | 0% |
| *20%* |  | | |  |  | 0% |
| *25%* |  | | |  |  | 0% |
| *30%* |  | | |  |  | 0% |
| *35%* |  | | |  |  | 0% |
| *40%* |  | | |  |  | 0% |
| *45%* |  | | |  |  | 0% |
| *50%* |  | | |  |  | 0% |
| *55%* |  | | |  |  | 0% |
| *60%* | *60%* | | | *100% (for performance range 60-100%)* | | ${p\left( B \right)n}_{6}= \frac{100\%-(50\%+2*13\%+2*8\%+3\%+2\%+2\%)}{9-6}*2$ *= 1.1%* |
| *65%* | *65%* | | |  |  | ${p\left( B \right)n}_{5}= \frac{100\%-(50\%+2*13\%+2*8\%+3\%+2\%)}{9-5}*2$ *= 1.7%* |
| *70%* | *70%* | | |  |  | ${p\left( B \right)n}_{4}= \frac{100\%-(50\%+2*13\%+2*8\%+3\%)}{9-4}*2$ *= 2.2%* |
| *75%* | *75%* | | |  |  | ${p\left( B \right)n}_{3}= \frac{100\%-(50\%+2*13\%+2*8\%)}{9-3}*2$ *= 2.8%* |
| *80%* | *80%* | | |  |  | ${p\left( B \right)n}_{2}= \frac{100\%-(50\%+2*13\%)}{9-(2*2-1)}*2$ *= 8.3%* |
| *85%* | *85%* | | |  |  | ${p\left( B \right)n}_{1}= \frac{100\%-50\%}{9-(2*1-1)}*2$ *= 12.5%* |
| *90%* | *90%* | | |  |  | $p_{mode}=$*50%* |
| *95%* | *95%* | | |  |  | ${p\left( B \right)n}_{1}= \frac{100\%-50\%}{9-(2*1-1)}*2$ *= 12.5%* |
| *100%* | *100%* | | |  |  | ${p\left( B \right)n}_{2}= \frac{100\%-(50\%+2*13\%)}{9-(2*2-1)}*2$ *= 8.3%* |

To generate a criterion performance that combined individual reviewer assessments, a performance distribution was constructed by:

1. assuming equal weighting of reviewer assessments
2. assuming a 100% probability that the combined performance falls within the reported range of likely performance across all reviewers assessing the proposal on the given criterion
3. randomly selecting one reviewer, and randomly selecting a performance estimate from their performance distribution, within a Monte Carlo framework

## Section 2. Decision trees to inform preference elicitation

This section presents the decision trees that were developed using a swing weighting algorithm to inform the elicitation of preferences related to: (1) partial values of *O1* and *O2*; (2) weights of relative importance between *O1* and *O2*; (3) time preference.

***Partial values of O1 and O2***

For each of *O1* and *O2*, SAC members answered up to six pairwise choice questions that iteratively approached this value mid-point. For instance, for *O1* the first question was:

*“Consider the following two proposals, each with different starting likelihoods of generating a vaccine that will be relevant for use in response to one of the CfP target pathogens. Imagine you are given the opportunity to improve the performance of one of these proposals. Which of the following options would you prefer?*

- *Option A: Improve Proposal A so that the likelihood that it generates a vaccine that will be relevant for use in response to one of the CfP target pathogens increases from 10% to x%*
- *Option B: Improve Proposal B so that the likelihood that it generates a vaccine that will be relevant for use in response to one of the CfP target pathogens increases from x% to 60%*
- *Indifferent between options A and B”*

In the first question, *x* was set as the mid-point in the performance range (35%). If a respondent was indifferent, the partial value function was considered linear, and no further questions were asked. If a respondent chose option A or option B, the value of *x* was updated according the logic defined in Figure 2.1, which presents the value of x for each survey iteration and the associated performance range used to calculate a depending on choice between options A, B, or indifference.

The pairwise choice questions identified *a* to be within a range. It was assumed that *a* was the mid-point in this range.

The partial value function for $O1$ and $O2$ was then defined by equations 4 and 5:

${PV}_{O1j}=\left\{ \begin{aligned} \left[ 0.5 . \left( \frac{{O1}_{i}}{a_{O1j}} \right) \right]. 100, if &{O1}_{i}<a_{O1j} \\ \left[ 0.5+0.5 . \left( \frac{{O1}_{i}-a_{O1j}}{{1- a}_{O1j}} \right) \right]. 100, if &{O1}_{i}\geq a_{O1j} \end{aligned} \right.$ (4)

${PV}_{O2j}=\left\{ \begin{aligned} \left[ 0.5 . \left( \frac{{O2}_{i}}{a_{O2j}} \right) \right]. 100, if &{O2}_{i}<a_{O2j} \\ \left[ 0.5+0.5 . \left( \frac{{O2}_{i}-a_{O2j}}{{1- a}_{O2j}} \right) \right]. 100, if &{O2}_{i}\geq a_{O2j} \end{aligned} \right.$ (5)

*Where:*

$a_{O1j}$ *= The value mid-point for* $O1$ *for survey respondent j*

$a_{O2j}$ *= The value mid-point for* $O2$ *for survey respondent j*

***Weights of relative importance between O1 and O2***

In order to elicit weights between *O1* and *O2*, the following question was asked:

*“Considering the following two proposals, which of these would you prefer?*

- *Proposal A:*
  - *Likelihood of generating a vaccine that will be relevant for use in response to one of the CfP1 target pathogens = y%*
  - *Likelihood that the technology will be suitable for use in vaccine development against newly emerging/unexpected pathogens = 10%*
- *Proposal B:*
  - *Likelihood of generating a vaccine that will be relevant for use in response to one of the CfP1 target pathogens = 10%*

*Likelihood that the technology will be suitable for use in vaccine development against newly emerging/unexpected pathogens = 60%”*

Figure 2.2 presents the value of y for each survey iteration and the performance range used at the end of six questions to calculate b depending on choice between options A or B.

Weights were then estimated for $O1$ and $O2$ for each SAC member (*j*) as described in equations 6 to 8:

$W_{O1j}= \frac{k_{j}}{1+ k_{j}}$ *(6)*

$W_{O2j}= \frac{1}{1+ k_{j}}$ *(7)*

*Where*

$k_{j}= \frac{60-10}{b-10}$ *(8)*

**Time preference**

In order to identify the value of c, such that SAC members were indifferent between a z% chance of successfully delivering a proposal within 5 years, and a 100% chance of doing so within 10 years, the following question was asked in several iterations:

*“Considering the following two proposals, which of these would you prefer?*

- *Proposal A:*
  - *Time-to-completion = 5 years*
  - *Likelihood of successful completion = z%*
- *Proposal B:*
  - *Time-to-completion = 10 years*
  - *Likelihood of successful completion = 100%*
- *Indifferent between Proposal A and Proposal B”*

Figure 2.3 presents the value of z for each survey iteration and the associated performance range used to calculate z depending on choice between options A, B, or indifference.

Given a value of c for survey respondent *j*, their discount rate was estimated using equation 9:

$r_{j}=({\frac{1}{c_{j}})}^{\frac{1}{5}}-1$ (9)

**Figure 2.1:** Levels used in choice questions to inform partial values of *O1,* *O2**

**If between iteration 1 and 6 a respondent chose indifference between options A and B, the performance range used to calculate a.is provided in [ ],*

**Figure 2.2:** Levels used in choice questions to inform *O1* and *O2* weights

**Figure 2.3:** Levels used in choice questions to inform time preferences*

**If between iteration 1 and 6 a respondent chose indifference between options A and B, the performance range used to calculate c.is provided in [ ],*
